# Supplementary material for: The Systems Biology Research Tool: evolvable open-source software
Source: BMC Syst Biol. 2008 Jun 29;2:55. doi: 10.1186/1752-0509-2-55 (PMC2446383; doi:10.1186/1752-0509-2-55)
Supplement: Additional file 1 — SBRT Archive. An archive of the current version of the Systems Biology Research Tool. [file 1752-0509-2-55-S1.zip › sbrt-1.4.0/doc/developers_guide/api/sbrt/kernel/fba/class-use/IrrevRxn.html]

Uses of Class sbrt.kernel.fba.IrrevRxn


|  |  |  |
| --- | --- | --- |
| **Overview** \| **Package** \| **Class** \| **Use** \| **Tree** \| **Deprecated** \| **Index** \| **Help** | |  |
| PREV   NEXT | **FRAMES**    **NO FRAMES**     **All Classes** |  |


---


## **Uses of Class sbrt.kernel.fba.IrrevRxn**

| Packages that use IrrevRxn | |
| --- | --- |
| **sbrt.kernel.fba** | Provides classes and interfaces for creating objects relevant to Flux Balance Analysis. |
| **sbrt.shell.text.chem** | Provides classes and interfaces for defining formats for chemistry related objects. |
| **sbrt.shell.text.fba** | Provides classes and interfaces for defining formats for objects relevant to Flux Balance Analysis. |

Uses of IrrevRxn in sbrt.kernel.fba

| Methods in sbrt.kernel.fba that return IrrevRxn | |
| --- | --- |
| `IrrevRxn` | `IrrevRxnFluxome.getInternalRxn(java.lang.String rxnName)`             Returns the internal reaction in this fluxome with the provided name. |
| `IrrevRxn` | `IrrevRxnFluxomeV1.getInternalRxn(java.lang.String reactionName)`             Returns the internal reaction in this fluxome with the provided name. |

| Methods in sbrt.kernel.fba that return types with arguments of type IrrevRxn | |
| --- | --- |
| `java.util.Map<java.lang.String,IrrevRxn>` | `IrrevRxnFluxome.getEquivRxns(java.lang.String reactionName)`             Returns the set reactions in this fluxome that are stoichiometrically equivalent to the provided reaction. |
| `java.util.Map<java.lang.String,IrrevRxn>` | `IrrevRxnFluxomeV1.getEquivRxns(java.lang.String rxnName)`             Returns the reactions in this fluxome that are stoichiometrically equivalent to the provided reaction. |
| `java.util.Map<java.lang.String,IrrevRxn>` | `IrrevRxnFluxome.getInternalRxns()`             Returns the set of all internal reactions in this fluxome. |
| `java.util.Map<java.lang.String,IrrevRxn>` | `IrrevRxnFluxomeV1.getInternalRxns()`             Returns all of the internal reactions in this fluxome. |
| `java.util.Map<java.lang.String,IrrevRxn>` | `IrrevRxnFluxome.getInternalRxns(java.util.Collection<java.lang.String> rxnNames)`             Returns the internal reactions with the provided names. |
| `java.util.Map<java.lang.String,IrrevRxn>` | `IrrevRxnFluxomeV1.getInternalRxns(java.util.Collection<java.lang.String> rxnNames)`             Returns the internal reactions whose names appear in the provided collection of reaction names. |
| `java.util.Map<java.lang.String,IrrevRxn>` | `IrrevRxnFluxomeV1.getRxns(java.lang.String catalyst)`             Returns all of the internal reactions contained in this fluxome that are catalyzed by the provided catalyst. |

Constructor parameters in sbrt.kernel.fba with type arguments of type IrrevRxn

`IrrevRxnFluxomeV1(java.util.Map<java.lang.String,IrrevRxn> internalRxns,
java.util.Map<java.lang.String,SourceRxn> sourceRxns,
java.util.Map<java.lang.String,SinkRxn> sinkRxns)`
  
          Constructs a new irreversible reaction fluxome from the
provided internal and exchange reactions.

`IrrevRxnFluxomeV1(java.util.Map<java.lang.String,IrrevRxn> internalRxns,
java.util.Map<java.lang.String,SourceRxn> sourceRxns,
java.util.Map<java.lang.String,SinkRxn> sinkRxns,
java.util.Map<java.lang.String,Or<And<java.lang.String>>> catalysts)`
  
          Constructs a new irreversible reaction fluxome from the
provided internal and exchange reactions and catalysts.

Uses of IrrevRxn in sbrt.shell.text.chem

| Methods in sbrt.shell.text.chem that return IrrevRxn | |
| --- | --- |
| `IrrevRxn` | `IrrevRxnFormatV2.parse(java.lang.String reaction)`             Parses the provided string and returns its corresponding irreversible reaction. |
| `IrrevRxn` | `IrrevRxnFormatV1.parse(java.lang.String reaction)`             Parses the provided string and returns its corresponding irreversible reaction. |

| Methods in sbrt.shell.text.chem with parameters of type IrrevRxn | |
| --- | --- |
| `java.lang.String` | `IrrevRxnFormatV2.format(IrrevRxn rxn)`             Returns a formatted string representation of the provided irreversible chemical reaction. |
| `java.lang.String` | `IrrevRxnFormatV1.format(IrrevRxn rxn)`             Returns a formatted string representation of the provided irreversible chemical reaction. |

Uses of IrrevRxn in sbrt.shell.text.fba

| Methods in sbrt.shell.text.fba with parameters of type IrrevRxn | |
| --- | --- |
| `java.lang.String` | `IrrevRxnFormatV1.format(IrrevRxn rxn)`             Returns a formatted string representation of the provided irreversible chemical reaction. |

---


|  |  |  |
| --- | --- | --- |
| **Overview** \| **Package** \| **Class** \| **Use** \| **Tree** \| **Deprecated** \| **Index** \| **Help** | |  |
| PREV   NEXT | **FRAMES**    **NO FRAMES**     **All Classes** |  |


---
